# Supplementary material for: Acute chemical ingestion in the under 19 population in South Korea: A brief report
Source: PLoS One. 2020 Nov 13;15(11):e0242430. doi: 10.1371/journal.pone.0242430 (PMC7665788; doi:10.1371/journal.pone.0242430)
Supplement: S1 Table — (DOCX) [file pone.0242430.s001.docx]

S1 Table. Total incidence and admission of chemical ingestion in children at ED by age group, South Korea, 2011-2016.

|  | Total | Age Group | | | Adm |  |  | Total | Age group | | | Adm |
| --- | --- | --- | --- | --- | --- | --- | --- | --- | --- | --- | --- | --- |
|  |  | ~5 Y | 6~12 Y | 13~19 Y |  |  |  |  | ~5 y | 6~12 y | 13~19 y |  |
| Acetic acid | 6 | 2 | 1 | 3 | 0 |  | Nickel | 1 | 1 | 0 | 0 | 0 |
| Acetone | 60 | 52 | 4 | 4 | 6 |  | Nicotine | 43 | 40 | 1 | 2 | 12 |
| Active carbon | 3 | 3 | 0 | 0 | 1 |  | Nitric acid | 1 | 0 | 0 | 1 | 0 |
| Ammonium chloride | 2 | 2 | 0 | 0 | 0 |  | Nitrogen | 6 | 4 | 1 | 1 | 0 |
| Ammonium phosphate | 4 | 0 | 2 | 2 | 1 |  | Paraffin | 5 | 5 | 0 | 0 | 1 |
| Arsenic | 2 | 0 | 0 | 2 | 0 |  | Phenolic Resin | 1 | 0 | 0 | 1 | 0 |
| Benzethonium chloride | 1 | 0 | 1 | 0 | 0 |  | Phenolphthalein | 1 | 1 | 0 | 0 | 0 |
| Benzoic acid | 1 | 1 | 0 | 0 | 0 |  | Phenoxyethanol | 4 | 4 | 0 | 0 | 0 |
| Boric acid | 14 | 14 | 0 | 0 | 4 |  | Phosphorus | 1 | 1 | 0 | 0 | 0 |
| Calcium carbonate | 1 | 1 | 0 | 0 | 0 |  | Polyester polyol | 1 | 0 | 1 | 0 | 0 |
| Calcium chloride | 17 | 16 | 0 | 1 | 0 |  | Polyethylene | 4 | 2 | 2 | 0 | 0 |
| Chlorine | 2 | 2 | 0 | 0 | 0 |  | Polyphenol | 4 | 4 | 0 | 0 | 0 |
| Citric acid | 47 | 47 | 0 | 0 | 3 |  | Polyvinyl alcohol | 2 | 1 | 0 | 1 | 0 |
| Copper sulfate | 2 | 0 | 2 | 0 | 1 |  | Potassium citrate | 1 | 1 | 0 | 0 | 0 |
| Diethylene glycol | 2 | 2 | 0 | 0 | 0 |  | Potassium cyanide | 1 | 0 | 0 | 1 | 0 |
| Diphenyl oxalate | 1 | 1 | 0 | 0 | 0 |  | Potassium dihydrogen phosphate | 1 | 0 | 0 | 1 | 0 |
| Epoxy | 3 | 2 | 0 | 1 | 0 |  | Potassium hydroxide | 1 | 0 | 1 | 0 | 0 |
| Ethanol | 141 | 116 | 1 | 24 | 8 |  | Silica | 2 | 2 | 0 | 0 | 0 |
| Ethyl cyanoacrylate | 9 | 7 | 1 | 1 | 1 |  | Silicagel | 55 | 52 | 2 | 1 | 0 |
| Ethylene glycol | 14 | 3 | 0 | 11 | 10 |  | Silicon | 2 | 2 | 0 | 0 | 0 |
| Formaldehyde | 1 | 0 | 0 | 1 | 0 |  | Sodium Carbonate | 1 | 1 | 0 | 0 | 0 |
| Gasoline | 2 | 2 | 0 | 0 | 1 |  | Sodium chlorite | 2 | 2 | 0 | 0 | 0 |
| Glacial acetic acid | 11 | 7 | 1 | 3 | 5 |  | Sodium fluoride | 3 | 3 | 0 | 0 | 0 |
| Glutaral | 1 | 0 | 0 | 1 | 1 |  | Sodium hydrogen carbonate | 6 | 6 | 0 | 0 | 0 |
| Glycerin | 14 | 14 | 0 | 0 | 0 |  | Sodium hydroxide | 65 | 57 | 6 | 2 | 5 |
| Gum arabic | 12 | 11 | 1 | 0 | 0 |  | Sodium hypochlorite | 247 | 141 | 24 | 82 | 64 |
| Heavy metal | 5 | 3 | 1 | 1 | 1 |  | Sodium lauroyl sarcosinate | 3 | 2 | 0 | 1 | 0 |
| Hydrochloric acid | 4 | 0 | 1 | 3 | 1 |  | Sodium lauryl sulfate | 14 | 12 | 1 | 1 | 1 |
| Hydrofluoric acid | 2 | 2 | 0 | 0 | 0 |  | Sodium metaphosphate | 3 | 1 | 0 | 2 | 0 |
| Hydrogen peroxide | 16 | 14 | 0 | 2 | 2 |  | Sodium nitrate | 1 | 0 | 0 | 1 | 0 |
| Hypochlorous acid | 2 | 1 | 1 | 0 | 0 |  | Sodium peroxocarbonate | 22 | 16 | 0 | 6 | 6 |
| Isopropyl alcohol | 2 | 2 | 0 | 0 | 2 |  | Sodium silicate | 3 | 3 | 0 | 0 | 0 |
| Kerosene | 8 | 5 | 1 | 2 | 2 |  | Sodium sulfite | 1 | 0 | 0 | 1 | 0 |
| Lactic acid | 2 | 2 | 0 | 0 | 0 |  | Stearic acid | 1 | 1 | 0 | 0 | 0 |
| Lauric acid | 6 | 6 | 0 | 0 | 0 |  | Sulfuric acid | 3 | 2 | 0 | 1 | 0 |
| Lithium | 2 | 2 | 0 | 0 | 0 |  | Surfactant | 203 | 171 | 9 | 23 | 17 |
| Magnesium chloride | 1 | 1 | 0 | 0 | 0 |  | Tea tree oil | 1 | 1 | 0 | 0 | 1 |
| Mercury[hydrargyrum] | 26 | 17 | 7 | 2 | 3 |  | Toluene | 16 | 12 | 1 | 3 | 4 |
| Methanol | 20 | 8 | 4 | 8 | 4 |  | Toluene Diisocyanate | 24 | 22 | 0 | 2 | 0 |
| Methyl acetate | 1 | 1 | 0 | 0 | 0 |  | Urea | 1 | 1 | 0 | 0 | 0 |
| Monoammonium phosphate | 1 | 1 | 0 | 0 | 0 |  | Varnish | 3 | 1 | 0 | 2 | 2 |
| Naphthalene | 16 | 14 | 2 | 0 | 3 |  |  |  |  |  |  |  |
|  |  |  |  |  |  |  | Total | 1247 | 959 | 80 | 208 | 173 |
|  |  |  |  |  |  |  |  | 100.0% | 76.9% | 6.4% | 16.7% | 13.9% |
